# Marked anti-tumour activity of the combination of YM155, a novel survivin suppressant, and platinum-based drugs

**DOI:** 10.1038/sj.bjc.6605713

**Published:** 2010-06-01

**Authors:** T Iwasa, I Okamoto, K Takezawa, K Yamanaka, T Nakahara, A Kita, H Koutoku, M Sasamata, E Hatashita, Y Yamada, K Kuwata, M Fukuoka, K Nakagawa

**Affiliations:** 1 Department of Medical Oncology, Kinki University School of Medicine, 377-2 Ohno-higashi, Osaka-Sayama, Osaka 589-8511, Japan; 2 Institute of Drug Discovery Research, Astellas Pharma Inc., 21 Miyukigaoka, Tsukuba-shi, Ibaraki 305-8585, Japan; 3 Department of Medical Oncology, Kinki University School of Medicine, Sakai Hospital, 2-7-1 Harayamadai, Minami-ku Sakai, Osaka 590-0132, Japan

**Keywords:** survivin, YM155, apoptosis, DNA repair, non-small cell lung cancer

## Abstract

**Background:**

Survivin, a member of the inhibitor of apoptosis protein family, is an attractive target for cancer therapy. We have now investigated the effects of the combination of YM155, a novel small-molecule inhibitor of survivin expression, and platinum compounds (cisplatin and carboplatin) on human non-small cell lung cancer (NSCLC) cell lines.

**Methods:**

The anti-cancer efficacy of YM155 in combination with platinum compounds was evaluated on the basis of cell death and progression of tumour xenografts. Platinum compound-induced DNA damage was evaluated by immunofluorescence analysis of histone *γ*-H2AX.

**Results:**

Immunofluorescence analysis of histone *γ*-H2AX showed that YM155 delayed the repair of double-strand breaks induced in nuclear DNA by platinum compounds. The combination of YM155 and platinum compounds also induced synergistic increases both in the number of apoptotic cells and in the activity of caspase-3. Finally, combination therapy with YM155 and platinum compounds delayed the growth of NSCLC tumour xenografts in nude mice to an extent greater than that apparent with either treatment modality alone.

**Conclusion:**

These results suggest that YM155 sensitises tumour cells to platinum compounds both *in vitro* and *in vivo*, and that this effect is likely attributable to the inhibition of DNA repair and consequent enhancement of apoptosis.

Survivin is a 16.5-kDa member of the inhibitor of apoptosis protein (IAP) family and blocks the mitochondrial pathway of apoptosis by inhibiting caspases (Altieri, 2003a, 2003b). Survivin also regulates cell division through interaction with the proteins INCENP and Aurora B (Wheatley *et al*, 2001). It is abundant in many types of cancer cells but not in the corresponding normal cells (Ambrosini *et al*, 1997; Marusawa *et al*, 2003; Altieri, 2004). High levels of survivin expression in cancer cells are associated with poor patient prognosis and survival, as well as with resistance to therapy and an increased rate of cancer recurrence (Monzo *et al*, 1999; Altieri, 2001; Rodel *et al*, 2005). Survivin has therefore become a therapeutic target and potentially important prognostic marker for many tumour types including non-small cell lung cancer (NSCLC) (Adida *et al*, 1998; Monzo *et al*, 1999). Reflecting the many mechanisms that seem to regulate survivin expression, diverse approaches have been evaluated for targeting survivin in experimental models (Li *et al*, 1999; Olie *et al*, 2000; Grossman *et al*, 2001).

YM155, a small imidazolium-based compound, was identified by high-throughput screening of chemical libraries for inhibitors of the activity of the survivin gene promoter in a reporter assay (Nakahara *et al*, 2007). This compound specifically inhibits the expression of survivin at both the mRNA and protein levels and exhibits pronounced anti-cancer activity in pre-clinical models (Nakahara *et al*, 2007). An advantage of YM155 compared with previously investigated suppressors of survivin expression is that it is active in the subnanomolar range (Carter *et al*, 2001; Milella *et al*, 2001; O’Connor *et al*, 2002; De Schepper *et al*, 2003; Wall *et al*, 2003; Sah *et al*, 2006). Our previous pharmacokinetics analysis also revealed that YM155 becomes highly distributed to tumour tissue in tumour xenograft models *in vivo* (Nakahara *et al*, 2007). In addition, continuous YM155 infusion in mice did not result in systemic toxicity such as body weight loss or decreased blood cell count (Nakahara *et al*, 2007). Furthermore, we have recently shown that YM155 sensitised NSCLC cells to radiation both *in vitro* and *in vivo*, and that this effect of YM155 was likely attributable to the inhibition of DNA repair and enhancement of apoptosis that result from downregulation of survivin expression (Iwasa *et al*, 2008). YM155 is thus an attractive candidate drug for cancer therapy.

Despite its demonstrated efficacy in targeting tumour cells, the effects of YM155 in combination with DNA-damaging drugs have remained largely unknown. We have now examined the effects of the combination of YM155 and platinum compounds on human NSCLC cell lines both *in vitro* and *in vivo*.

## Materials and methods

### Cell culture and reagents

The human NSCLC cell lines NCI-H460 (H460), Calu6, NCI-H358 (H358), and PC14 were obtained from the American Type Culture Collection (Manassas, VA, USA). The cells were cultured under an atmosphere of 5% CO_2_ at 37°C in RPMI 1640 medium (Sigma, St Louis, MO, USA) supplemented with 10% foetal bovine serum. Cisplatin (CDDP) was obtained from Nippon Kayaku (Tokyo, Japan), and carboplatin (CBDCA) was from Bristol-Myers Squibb (New York, NY, USA). YM155 (Astellas Pharma Inc, Tokyo, Japan) was dissolved in dimethyl sulfoxide (DMSO).

### Immunoblot analysis

Cells were washed twice with ice-cold phosphate-buffered saline (PBS) and then lysed in a solution containing 20 mM Tris–HCl (pH 7.5), 150 mM NaCl, 1 mM EDTA, 1% Triton X-100, 2.5 mM sodium pyrophosphate, 1 mM phenylmethylsulfonyl fluoride, and leupeptin (1 *μ*g ml^−1^). The protein concentration of lysates was determined with the Bradford reagent (Bio-Rad, Hercules, CA, USA), and equal amounts of protein were subjected to SDS polyacrylamide gel electrophoresis on a 15% gel. The separated proteins were transferred to a nitrocellulose membrane, which was then exposed to 5% non-fat dried milk in PBS for 1 h at room temperature before overnight incubation at 4°C with rabbit polyclonal antibodies to human survivin (1 : 1000 dilution; R&D Systems, Wiesbaden, Germany), to human c-IAP1 (1:1000 dilution; MBL International, Woburn, MA, USA), or to human XIAP (1:1000 dilution; Cell Signaling, Beverly, MA). The membrane was then washed with PBS containing 0.05% Tween 20 before incubation for 1 h at room temperature with horseradish peroxidase-conjugated goat antibodies to rabbit immunoglobulin G (Sigma). Immune complexes were finally detected with chemiluminescence reagents (PerkinElmer Life Science, Boston, MA, USA).

### Detection of apoptotic cells

Cells were fixed with 4% paraformaldehyde for 1 h at room temperature, after which a minimum of 1000 cells per sample was evaluated for apoptosis with the use of the terminal deoxynucleotidyl transferase-mediated dUTP nick-end labelling (TUNEL) technique (*In situ* Cell Death Detection Kit; Boehringer Mannheim, Mannheim, Germany).

### Assay of caspase-3 activity

The activity of caspase-3 in cell lysates was measured with a CCP32/caspase-3 Fluometric Protease Assay Kit (MBL). Fluorescence attributable to cleavage of the DEVD-AFC substrate was measured at excitation and emission wavelengths of 390 and 460 nm, respectively.

### Immunofluorescence staining of *γ*-H2AX

Cells were grown to 50% confluence in two-well Lab-Tec Chamber Slides (Nunc, Naperville, IL, USA) and then cultured for 48 h in the presence of 50 nM YM155 or vehicle (final DMSO concentration of 0.1% we confirmed that this DMSO concentration did not affect the proliferation of NSCLC cell lines) before additional exposure to 10 *μ*M CDDP or CBDCA. At various times thereafter, the cells were fixed with 4% paraformaldehyde for 10 min at room temperature, permeabilised with 0.1% Triton X-100 for 10 min at 4°C, and exposed to 5% non-fat dried milk for 10 min at room temperature. The slides were washed with PBS and then incubated at room temperature first for 2 h with mouse monoclonal antibodies to histone *γ*-H2AX (Upstate Biotechnology, Lake Placid, NY, USA) at a dilution of 1 : 300 and then for 1 h with Alexa Fluor 488-labeled goat antibodies to mouse immunoglobulin G (Molecular Probes, Eugene, OR, USA) at a dilution of 1 : 700. The slides were mounted in fluorescence mounting medium (Dako Cytomation, Hamburg, Germany), and fluorescence signals were visualised with a confocal laser-scanning microscope (Axiovert 200M; Carl Zeiss, Oberkochen, Germany) equipped with the LSM5 PASCAL system (Carl Zeiss). Three random fields each containing at least 50 cells were examined at a magnification of × 100. Nuclei containing ⩾10 immunoreactive foci were counted as positive for *γ*-H2AX, and the percentage of positive cells was calculated (De Schepper *et al*, 2003).

### Evaluation of tumour growth *in vivo*

Male nude (BALB/cAnNCrj-nu/nu) mice (5 weeks old) were obtained from Charles River Japan (Kanagawa, Japan). All animal studies were performed in accordance with the Recommendations for Handling of Laboratory Animals for Biomedical Research compiled by the Committee on Safety and Ethical Handling Regulations for Laboratory Animal Experiments, Kyoto University. The ethical procedures followed met the requirements of the UKCCCR guidelines (1998). Tumour cells (2 × 10^6^) were implanted into the right hind leg of 6-week-old male athymic nude mice. Tumour volume was determined from caliper measurement of tumour length (*L*) and width (*W*) according to the formula *LW*^2^/2. Treatment was initiated when tumours in each group of animals achieved an average volume of 100–200 mm^3^. Treatment groups (each containing eight mice) consisted of vehicle control (0.1% DMSO in physiological saline), YM155 alone, vehicle plus CDDP or CBDCA, and YM155 plus CDDP or CBDCA. Vehicle or YM155 at a dose of 5 mg per kg of body mass was administered over seven consecutive days (days 0–6) with the use of an implanted micro-osmotic pump (Alzet model 1007D; Durect, Cupertino, CA, USA). CDDP (3 mg kg^−1^) or CBDCA (60 mg kg^−1^) was administered intravenously on each of days 0–3 and days 7–11, respectively.

### Statistical analysis

Data are presented as means±s.e. and were compared between groups with the unpaired Student's *t*-test. A *P*-value of <0.05 was considered statistically significant.

## Results

### Specific inhibition of survivin expression in NSCLC cells by YM155

**Figure 1:**
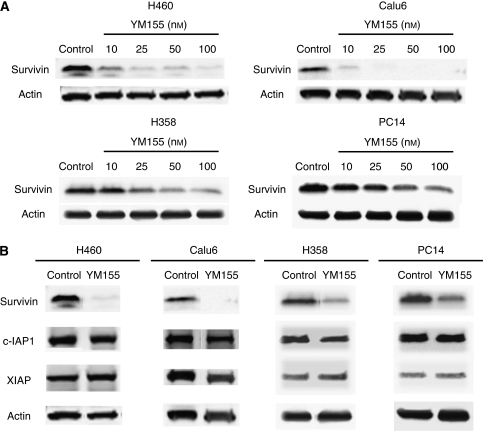
Effect of YM155 on survivin expression in human non-small cell lung cancer (NSCLC) cells. (**A**) H460, Calu6, H358, or PC14 cells were incubated in the absence (control, 0.1% dimethyl sulfoxide (DMSO)) or presence of the indicated concentrations of YM155 for 48 h. Cell lysates were then prepared and subjected to immunoblot analysis with antibodies to survivin or to *β*-actin (loading control). (**B**) H460, Calu6, H358, or PC14 cells were incubated in the absence or presence of 50 nM YM155 for 48 h, after which cell lysates were subjected to immunoblot analysis with antibodies to survivin, c-IAP1, XIAP, or *β*-actin.

We first examined the effect of YM155 on survivin expression in human NSCLC cell lines by immunoblot analysis. Exposure of H460, Calu6, H358, or PC14 cells to YM155 at 10–100 nM for 48 h inhibited survivin expression in a concentration-dependent manner (Figure 1A). In contrast, exposure of these cell lines to YM155 at 50 nM for 48 h did not affect the abundance of other members of the IAP family including XIAP and c-IAP1 (Figure 1B), indicating that YM155 specifically inhibits survivin expression in the NSCLC cell lines.

### Enhancement of DNA-damaging agent-induced apoptosis in NSCLC cells by YM155

**Figure 2:**
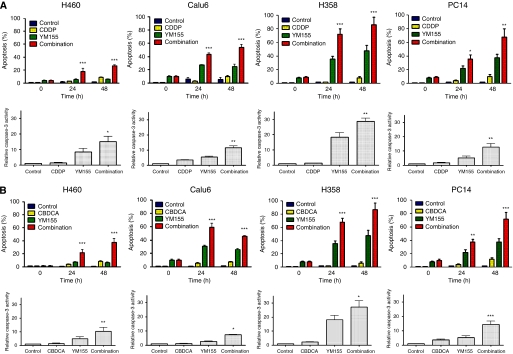
Effects of YM155 on DNA-damaging agent-induced apoptosis and caspase-3 activity in H460, Calu6, H358, or PC14 cells. Cells were incubated with 50 nM YM155 or vehicle (0.1% dimethyl sulfoxide (DMSO)) for 48 h and then for the indicated times (upper panels) or for 24 h (lower panels) in the additional absence or presence of 10 *μ*M CDDP (**A**) or CBDCA (**B**). The percentage of apoptotic cells was then determined by terminal deoxynucleotidyl transferase-mediated dUTP nick-end labeling (TUNEL) staining (upper panels), and cell lysates were assayed for caspase-3 activity (lower panels). All data are means±s.e. from three independent experiments; those for caspase-3 activity are expressed relative to the corresponding value for the control condition. ^*^P<0.05, ^**^P<0.001, ^***^P<0.0001 *vs* the corresponding value for CDDP or CBDCA or for YM155 alone.

We next examined the effect of YM155 on DNA-damaging agent-induced apoptosis in H460, Calu6, H358, or PC14 cells with the use of the TUNEL assay. Combined treatment of each cell line with YM155 and either CDDP or CBDCA resulted in an increase in the number of apoptotic cells at 24 and 48 h that was greater than the sum of the increases induced by YM155 or by CDDP or CBDCA alone (Figure 2). To confirm the results of the TUNEL assay, we measured the activity of caspase-3 in cell lysates. Again, combined treatment of H460, Calu6, H358, or PC14 cells with YM155 and either CDDP or CBDCA induced a synergistic increase in caspase-3 activity (Figure 2). These data thus suggested that YM155 promotes the induction of apoptosis by DNA-damaging agents in NSCLC cell lines.

### Effect of YM155 in combination with DNA-damaging agents on H2AX phosphorylation

**Figure 3:**
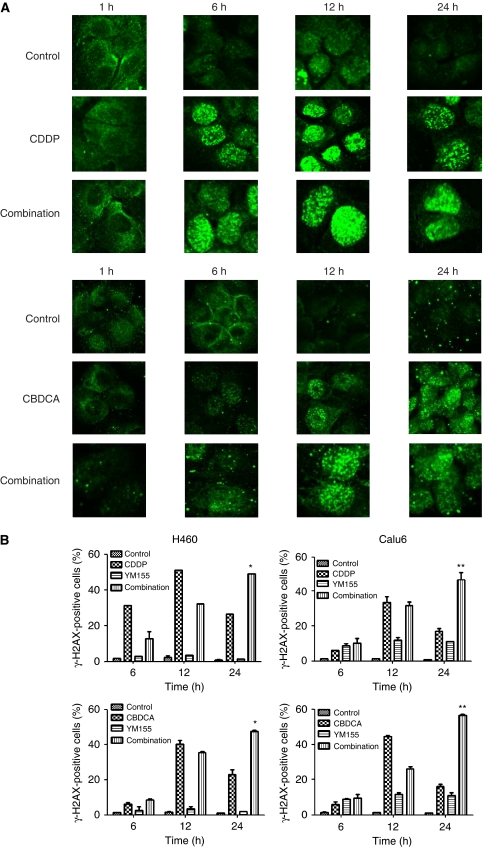
Effect of YM155 on the formation of *γ*-H2AX foci induced by DNA-damaging agents in non-small cell lung cancer (NSCLC) cells. (**A**) H460 cells were incubated with vehicle (0.1% dimethyl sulfoxide (DMSO)) or 50 nM YM155 for 48 h and then for the indicated times in the additional absence or presence of 10 *μ*M CDDP or CBDCA . The cells were then fixed and subjected to immunofluorescence staining for *γ*-H2AX (green fluorescence). (**B**) H460 or Calu6 cells were incubated with vehicle or 50 nM YM155 for 48 h and then for the indicated times in the additional absence or presence of 10 *μ*M CDDP or CBDCA . They were then fixed and subjected to immunofluorescence staining for *γ*-H2AX, and the percentage of cells containing *γ*-H2AX foci was determined. Data are means±s.e. from three independent experiments. ^*^P<0.05, ^**^P<0.001 *vs* the corresponding value for CDDP or CBDCA alone.

We have previously shown that YM155 sensitises tumour cells to radiation by inhibiting the repair of radiation-induced DNA damage. CDDP and CBDCA are key drugs in NSCLC treatment and are known to induce DNA damage (Diggle *et al*, 2005). We therefore hypothesised that YM155 might inhibit the repair of CDDP- or CBDCA-induced DNA damage and thereby promote CDDP- or CBDCA-induced cell death. To explore this possibility, we determined whether YM155 might affect CDDP- or CBDCA-induced phosphorylation of histone H2AX to yield *γ*-H2AX, which is a marker of DNA double-strand breaks (DSBs). Exposure of H460 or Calu6 cells to CDDP or CBDCA resulted in the gradual accumulation of *γ*-H2AX foci, with this effect being maximal at 12 h, after which the number of foci declined (Figure 3). Although YM155 did not affect the extent of CDDP- or CBDCA-induced focus formation, it significantly retarded the loss of foci normally apparent at 24 h after exposure to CDDP or CBDCA (Figure 3). These results thus suggested that downregulation of survivin expression by YM155 results in inhibition of the repair of DSBs induced by DNA-damaging agents in NSCLC cells.

### Enhancement of chemotherapy-induced tumour regression by YM155

**Figure 4:**
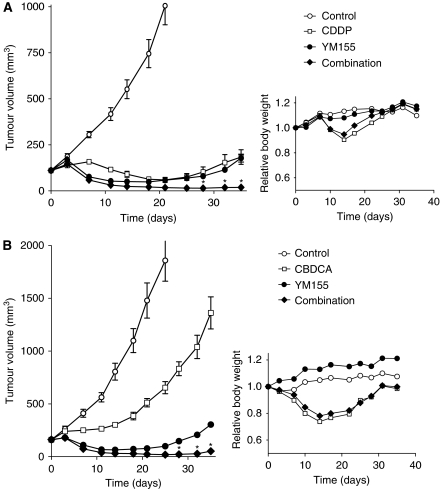
Effects of YM155 on the anti-tumour action of CDDP or CBDCA *in vivo*. Calu6 cells were injected into the right hind limb of nude mice and allowed to form tumours, after which the mice were assigned to one of four treatment groups (control, CDDP (**A**) or CBDCA (**B**) alone, YM155 alone, or the combination of YM155 and either CDDP (**A**) or CBDCA (**B**)) as described in Materials and Methods. Tumour volume was measured at the indicated times after the onset of treatment (left panels); values for mice in the control group are not shown for later time points to highlight differences among the other three groups. Body weight was also measured in each treatment group at the indicated times and is expressed relative to the corresponding value for time 0 (right panels). All data are means±s.e. from eight mice per group. ^*^P<0.0001 *vs* the corresponding value for treatment with CDDP (**A**) or CBDCA (**B**) alone or with YM155 alone.

To determine whether the enhancement of the anti-tumour activity of DNA-damaging agents by YM155 observed *in vitro* might also be apparent *in vivo*, we injected Calu6 cells into nude mice to elicit the formation of solid tumours. After tumour formation, the mice were treated with YM155, CDDP, or both drugs. Combined treatment with CDDP and YM155 inhibited Calu6 tumour growth to a markedly greater extent than did treatment with either drug alone (Figure 4A). No pronounced tissue damage or toxicity such as weight loss (Figure 4A) was observed in mice in any of the four treatment groups.

Finally, we examined the effect of the combination of YM155 and CBDCA on tumour growth. Treatment with YM155 or CBDCA alone delayed tumour growth, whereas combined treatment inhibited tumour growth to a significantly greater extent (Figure 4B). Again, there was no evidence of toxicity on the basis of body weight loss (Figure 4B) and there were no animal deaths in any of the four groups. These data suggested that YM155 enhances the tumour response to platinum-based chemotherapy *in vivo*.

## Discussion

The success of anti-cancer therapies is often limited by the development of resistance to apoptosis, which may result from defects in common apoptotic pathways (Hanahan and Weinberg, 2000). In this context, approaches to counteract the action of survivin in tumour cells have been proposed with the dual aims of inhibiting tumour growth through promotion of spontaneous apoptosis and of enhancing the tumour cell response to apoptosis-inducing agents (Altieri, 2003b). In this study, we found that the combination of YM155 and platinum compounds induced NSCLC cell apoptosis as well as the activation of caspase-3 to an extent greater than that apparent with either type of agent alone. Our findings thus suggest that YM155 acts in a synergistic manner to promote the induction of apoptosis by platinum compounds.

Cellular responses to stress or DNA damage are important for the maintenance of genomic stability and cellular integrity (Bunz *et al*, 1998; Hirao *et al*, 2000). Depending on its extent, cells either repair DNA damage or, when it is too severe for repair, initiate the cell death programme (Zhao *et al*, 2001). Agents that inhibit repair of DNA damage therefore increase the sensitivity of cells to ionising radiation and chemotherapeutic drugs (Banath *et al*, 2004; Taneja *et al*, 2004). The chemotherapeutic effect of platinum compounds results from their interaction with DNA; platinum thus induces the formation of DNA–protein crosslinks, DNA monoadducts, as well as interstrand or intrastrand DNA crosslinks (Chu, 1994; Siddik, 2003). These DNA adducts induce local distortion in the DNA double helix that results in strand unwinding and kinking (Chu, 1994). Survivin was previously shown to enhance tumour cell survival after radiation exposure through regulation of DSB repair (Chakravarti *et al*, 2004). We have previously shown that YM155 inhibited the repair of radiation-induced DSBs in NSCLC cells and that this effect likely accounted for the observed radiosensitising action of YM155 (Iwasa *et al*, 2008). We therefore investigated the effect of YM155 on the repair of platinum compound-induced DSBs by immunofluorescence imaging of *γ*-H2AX foci. Given that *γ*-H2AX appears rapidly at DNA DSBs and disappears as repair proceeds (Rogakou *et al*, 1998; Zhou and Elledge, 2000; Khanna *et al*, 2001; Shiloh and Kastan, 2001), it serves as a sensitive and specific marker for unrepaired DNA damage. We found that YM155 inhibited the repair of platinum compound-induced DSBs in NSCLC cells. Overexpression of survivin was previously shown to enhance DSB repair in tumour cells through upregulation of Ku protein (Jiang *et al*, 2009). Although it remains unclear whether YM155 affects Ku protein kinetics, our data suggest that the observed chemosensitisation by YM155 is attributable to inhibition of the repair of DNA damage induced by CDDP or CBDCA. Further investigations will be required to determine the mechanism underlying the effect of YM155 on DNA repair.

In addition to the initially identified isoform, four splice variants of human survivin have been described: survivin-2*α*, survivin-2*β*, survivin-*δ*-Ex3, and survivin-3*β* (Mahotka *et al*, 1999, 2002; Badran *et al*, 2004; Caldas *et al*, 2005a). However, little is known of the differential functions of these alternative splice forms of survivin (Krieg *et al*, 2002; Mahotka *et al*, 2002; Caldas *et al*, 2005b; Noton *et al*, 2006). Given that the suppression of survivin expression by YM155 is mediated through inhibition of the transcriptional activity of the survivin gene promoter (Nakahara *et al*, 2007), it is possible that YM155 also inhibits the expression of these survivin variants.

No serious adverse haematological events related to drug treatment were reported in phase I studies of YM155 in single-agent therapy (Tolcher *et al*, 2008; Satoh *et al*, 2009). A recent phase II study showed that YM155 monotherapy is safe but only moderately effective (objective tumour response rate, 5.4%) in patients with advanced NSCLC (Giaccone *et al*, 2009). Given this limited efficacy of YM155 as a single agent in NSCLC patients, the combination of YM155 with other agents may be beneficial. Platinum-based combination chemotherapy is the standard of care for most individuals with advanced NSCLC (Chu, 1994). We have shown that treatment of NSCLC cells with YM155 results in a marked increase in the anti-tumour effects of CDDP and CBDCA both *in vitro* and *in vivo*, suggesting that the combination of YM155 and platinum compounds may have potential as a novel therapeutic regimen. Clinical studies of YM155 in combination with platinum-based chemotherapy are thus warranted.
